# Feasibility study for early supported discharge in adults with respiratory infection in the UK

**DOI:** 10.1186/1471-2466-14-25

**Published:** 2014-02-26

**Authors:** Andrea M Collins, Odiri J Eneje, Carole A Hancock, Daniel G Wootton, Stephen B Gordon

**Affiliations:** 1 Biomedical Research Centre (BRC) in Microbial Diseases, Respiratory Infection Group, Royal Liverpool and Broadgreen University Hospital Trust, Prescot Street, L7 8XP Liverpool, UK; 2 Respiratory Infection Group, 3rd Floor Liverpool School of Tropical Medicine, Pembroke Place L3 5QA Liverpool, UK; 3 Department of Respiratory Research, University Hospital Aintree, Longmoor Lane L9 7AL Liverpool, UK

**Keywords:** Early supported discharge, Pneumonia, Respiratory infection, Feasibility, Patient acceptability

## Abstract

**Background:**

Many patients with pneumonia and lower respiratory tract infection that could be treated as outpatients according to their clinical severity score, are in fact admitted to hospital. We investigated whether, with medical and social input, these patients could be discharged early and treated at home.

*Objectives*: (1) To assess the feasibility of providing an early supported discharge scheme for patients with pneumonia and lower respiratory tract infection (2) To assess the patient acceptability of a study comprising of randomisation to standard hospital care or early supported discharge scheme.

**Methods:**

*Design*: Randomised controlled trial.

*Setting*: Liverpool, UK. Two University Teaching hospitals; one city-centre, 1 suburban in Liverpool, a city with high deprivation scores and unemployment rates.

*Participants*: 200 patients screened: 14 community-dwelling patients requiring an acute hospital stay for pneumonia or lower respiratory tract infection were recruited.

*Intervention*: Early supported discharge scheme to provide specialist respiratory care in a patient’s own home as a substitute to acute hospital care.

*Main outcome measures*: Primary - patient acceptability. Secondary – safety/mortality, length of hospital stay, readmission, patient/carer (or next of kin) satisfaction, functional status and symptom improvement.

**Results:**

42 of the 200 patients screened were eligible for early supported discharge; 10 were only identified at the point of discharge, 18 declined participation and 14 were randomised to either early supported discharge or standard hospital care. The total hospital length of hospital stay was 8.33 (1–31) days in standard hospital care and 3.4 (1–7) days in the early supported discharge scheme arm. In the early supported discharge scheme arm patient carers reported higher satisfaction with care and there were less readmissions and hospital-acquired infections.

*Limitations*: A small study in a single city. This was a feasibility study and therefore not intended to compare outcome data.

**Conclusions:**

An early supported discharge scheme for patients with pneumonia and lower respiratory tract infection was feasible. Larger numbers of patients would be eligible if future work included patients with dementia and those residing in care homes.

**Trial registration:**

ISRCTN25542492.

## Background

Large variability in rates of hospitalisation for patients with pneumonia and lower respiratory tract infection (LRTI) exists across nearby geographical regions. Commentators suggest that criteria for determining hospital admission and length of stay (LOS) are uncertain, physician-dependent and influenced by to socio-economic status and social support [1-3]. Seventy percent of UK pneumonia admissions are for patients with low-risk pneumonia (CURB-65 score 0–2) [4]; guidelines suggest that these patients do not require admission however these patients account for a significant proportion of bed days and costs [1]. Often, factors other than disease severity prompt or prolong hospital admission such as the inability to cope at home alone or to tolerate oral antibiotics, co-morbid illnesses, homelessness and substance abuse [5,6]_._ With the provision of medical support at home many more patients could be managed as outpatients [7]. In Europe and the USA, 57% and 90% of pneumonia/CAP (respectively) expenditure relates to the cost of in-patient care [8,9]. Reduction of this resource burden is an international priority.

For the elderly, in particular, hospital admission may not only be unnecessary but also more detrimental compared to care in their own residence, by increasing the risk of confusion and hospital-acquired infection (HAI) such as hospital acquired pneumonia (HAP) [10]. It is therefore important to specifically address care provision for elderly patients with respiratory tract infection by providing the option of Hospital at Home (HAH).

HAH is defined as a service where active treatment is provided by healthcare professionals in the patient’s home for a condition that otherwise would require acute hospital in-patient care, for a limited time period [11]. HAH schemes may aim for *admission avoidance (AA)* [avoiding hospital admission altogether] and/or *early supported discharge (ESD)* [discharging patients from hospital earlier than standard hospital care (SHC) and thereby reducing length of stay (LOS)].

Evidence of benefit in both AA and ESD schemes exists in chronic obstructive pulmonary disease (COPD) [12-19]. The evidence base for HAH schemes in pneumonia and LRTI is very limited. A recent expert review suggested that supported home care for patients with CAP ‘shows enormous potential for improving the care of elderly and disabled patients, and should be further evaluated in terms of efficacy and cost-effectiveness’ [20].

We prospectively studied the feasibility of a randomised controlled study of an ESDS for patients with pneumonia and LRTI: *HOME F*ollowed-up with *I*nfection *R*espiratory *S*upport *T*eam (*HOME FIRST*).

## Methods

We carried out a randomised feasibility study of an early supported discharge scheme (ESDS) versus standard hospital care (SHC) for patients admitted to hospital with pneumonia or LRTI. SHC in our city-centre teaching hospital consists of patients being admitted through the emergency department (self-presenting) or directly to the acute medical admissions unit (AMAU) via their GP. All patients that are to remain inpatients then stay on AMAU for at least 12 hours in general prior to ward transfer. On the AMAU the patient is clerked by a junior doctor on the on-call team (this may be a foundation year [FY] 1, 2, core medical trainee [CMT] or specialist registrar). The patient is then reviewed by an acute medical consultant within 12 hours on the post-take ward round on AMAU prior to transfer to the medical ward; this may be a general medical, respiratory or infectious disease ward, depending on bed capacity. After this the number and seniority of reviews differs per ward but in general consultant wards rounds occur 2 – 3 times weekly and registrar ward rounds once to twice weekly, the patient is reviewed on a daily basis on week days by a FY1, 2 or CMT trainee. Patients are referred to respiratory medicine for specialist opinion as deemed necessary by their team.

### Eligibility criteria

Patients ≥18 yrs old, admitted to hospital for pneumonia or LRTI from January - April 2012 were considered for recruitment. All CURB-65 scores were considered. In order to participate, patients were required to meet study eligibility criteria and provide written informed consent. These criteria were designed to identify patients suitable for this type of intervention (Table 1).

**Table 1:** Selection criteria

**Patient eligibility**	
Patients with any of the following conditions:	• Pneumonia – CAP or HAP [radiological consolidation and symptoms/signs of respiratory infection] N.B. if CURB-65 ≥ 3 MUST have had at least 24hrs of in-patient observation before recruitment.
	• Non-pneumonic lower respiratory tract infection [No radiological consolidation but symptoms/signs of respiratory infection]
	• Pneumonia with concomitant COPD (if this service is not provided elsewhere)
**Inclusion criteria**	
Features on history	• Patient able to give fully informed consent
	• Has a phone
	• Age > 18yrs old
Features on examination (stability indicator)	• Early warning score ≤2 (EWS, a score calculated using baseline observations) *AND* SBP > 90 *AND* mild confusion only (Abbreviated mini-mental test score [AMTS] ≥ 7). All observations must be stable for 12-24hrs
	• Stable/improving inflammatory markers (WCC/CRP)
	• Stable/improving U&Es
Features of social situation	• Can manage ADLs with current support (immediate OT/physiotherapy/social care can be arranged)
**Exclusion criteria**	
Features on history	• Well enough for discharge without home care support
	• No fixed abode
Features on examination (instability indicator)	• SBP < 90 mmHg
	• For patients with chronic respiratory illness: saturations <88% on air [except asthma]
	• For patients without chronic respiratory illness: saturations <92% on air
Features of diagnosis (indicating cause for concern)	• Suspected MI/raised TnI/T consistent with NSTEMI within 5 days of discharge
	• Empyema or complicated parapneumonic effusion
	• Tuberculosis suspected
	• Neutropenia
	• Acute exacerbations of COPD – infective & non-infective (other services are already provided)
	• Serious co-morbidities requiring hospital treatment (e.g: CKD, CCF) or deemed unstable (significant AKD)
Features of social situation	• Patients unable to manage at home even with maximal support (e.g. IV drug users, alcohol excess or mental health problems)

### Randomisation and approval

Subjects were randomly assigned using computer generated random numbers to receive either ESDS or SHC. Allocation was obtained by telephoning an independent co-ordinator (closed envelope system). The local NHS Research and Ethics Committee (REC North-West Liverpool Central [11/NW/0670]) granted approval for the study which was sponsored by Royal Liverpool and Broadgreen Hospital trust (RLBUHT) and University Hospital Aintree (UHA).

### Study sites

The study was conducted at 2 sites, 1 city-centre University hospital (RLBUHT – 710 beds), one suburban University hospital (UHA – 743 beds) both within the same city.

### Study intervention

We offered early supported discharge by providing specialist respiratory care to patients in their own home to substitute acute hospital care. This care was provided by an experienced hospital respiratory doctor and nurse team who provided up to twice daily direct care and were able to perform blood tests, observations and clinical examinations. Oxygen [O_2_] (if not already receiving domiciliary O_2_), intravenous (IV) fluids and IV antibiotics were not provided. The patient was followed by the same study doctor until stable for discharge from the ESDS, after this, care was provided by their general practitioner as usual. Fast-access to discharge medications, a disease-specific patient information leaflet and ‘meals-on-wheels’ (ready-made food delivery service) were provided as required. SHC in our hospitals comprises of both systematic, and as required, medical review.

### Screening and recruitment

Potentially eligible patients were identified using a standard protocol. Only patients who would have required ‘at least one more night of hospitalisation before discharge’ were considered. We hypothesised that various reasons for this continued hospitalisation may exist, since there is no specific guidance as to when a patient recovering from LRTI is suitable for discharge and therefore inter-physician variability exists. Where the study doctor considered a patient well enough for discharge without support, the usual medical team were notified. Subjects were randomised to either SHC or ESDS. Age, gender and reason(s) for a lack of eligibility/suitability were noted for all screened patients. Patients already on home O_2_ therapy were included in the study if their saturations were >87% on their usual Fi0_2_.

Recruited subjects provided a clinical history, were examined by the study doctor and completed an SF-12 questionnaire [21] (functional and quality of life assessment tool) at day 0 and two CAP-SYM questionnaires [22] (symptom score) for day 0 and day ‘minus 30’ (the patient was asked to recall their symptoms from 30 days prior to study recruitment). Nasal wash, serum, sputum, blood cultures, clinical bloods and urine were obtained at day 0. SF-12, CAP-SYM, nasal wash and serum (+/− clinical bloods as needed) were performed on day 2 and 7; for patients who had been discharged, these investigations were performed in their home.

Subjects in the ESDS arm were transferred home the same day with appropriate medications, an emergency 24 hr contact telephone number, a list of symptoms to prompt healthcare contact (fever > 38° Celsius, increasing drowsiness, worsening cough or sputum and/or increasingly unwell) and an observations machine capable of recording temperature, BP, HR and O_2_ saturations. If the discharge was before 3 pm the subject was reviewed at home later that evening by the team; if after 3 pm the review was the next morning. The frequency and duration of home visits was determined by communication between the medical team, patient and carer/next of kin. Telephone calls were used instead of home visits where the study team felt this suitable. Each visit lasted between 10-30mins. During home visits the following were recorded - BP, HR, O_2_ saturations and temperature (on an observations form), clinical symptoms and examination findings, ability to eat/drink and appetite, bowel habit, and current mobility/exercise tolerance. Ability to cope at home and medication concordance were assessed. Any evidence of confusion was thoroughly assessed using the AMTS. Smoking advice was offered and new issues, problems and symptoms were addressed. The case report form provided a guide for recognising patients who needed consideration for readmission, using a simple set of clinical and functional questions. Reasons for considering a patient suitable for discharge from HOME FIRST included:

● Resolution of the reason for continued hospitalisation

● Temp <37.5 degrees Celsius

● BP > 90 mm Hg

● Saturations > 86% on oxygen or 90% on air

● 50% reduction in highest CRP (unless non-infective reason for high CRP)

● Stable non-pneumonic co-morbidities (patient handed over to community team if further follow-up needed)

● Able to manage with current care level

Reasons for readmission to hospital included:

● Social concern

● Reduced eating & drinking

● Fall

● Increasing CRP/WCC

● Unable to take antibiotics

● Oxygen saturations drop >2%

● RR rise ≥10

● Temp ≥38

● GCS drop ≥ 2

● No PU > 12 hrs

● Any other cause of clinical concern

Two weeks after recruitment all subjects and their next of kin/carer received a telephone call from an independent assessor to complete a care satisfaction questionnaire (see Additional file 1). All subjects were asked to attend an outpatient appointment at 1 and 6 months post recruitment; a clinical assessment, CAP-SYM, SF-12 and bloods (including serum) were performed.

### Outcomes and operational questions

This was a feasibility study with a primary outcome of patient acceptability to randomisation. Data collected as secondary outcomes included safety/mortality, patient/carer satisfaction, readmission rates, total hospital LOS/days of care, functional status/quality of life and symptom improvement. Cost was not assessed.

### Safety

An experienced specialist respiratory doctor (a senior respiratory registrar with more than 10 years of clinical experience) and respiratory nurses (band 6) with ward and community experience used strict patient selection criteria (see Table 1) such as ‘must have a telephone’, ‘must be able to manage at home’ and minimum saturation and BP thresholds to ensure patient safety. Subjects received through education and a detailed information leaflet with ‘red-flag’ symptoms (see Additional file 2). The study team provided regular home visit and a 24 hr telephone on-call service. Fast tracked re-admission was arranged if deemed necessary.

### Sample size and statistical methods

Our sample size was pragmatic allowing recruitment in a single winter season. We planned to screen a minimum of 100 patients, planning to recruit 20 subjects.

## Results

**Figure 1:**
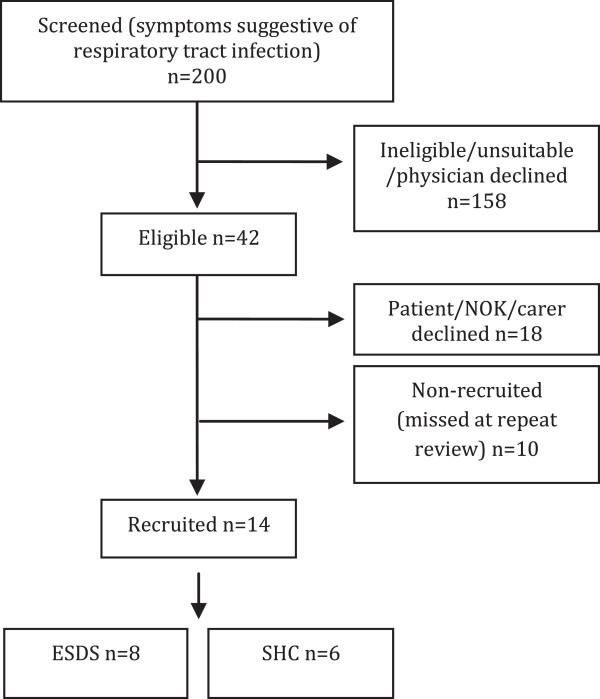
**Screening and final recruitment numbers.** Note no patients withdrew consent or were lost to follow-up. ‘Missed’ means missed due to logistical reasons e.g. by the time of repeat patient review by the study team the patient was well enough for discharge without ESDS support or the patient was discharged outside of the hours/days of study recruitment.

During the 4-month study period 200 patients (with symptoms suggestive of respiratory infection) were screened. 158 were ineligible (see Table 2). Of the 42 eligible patients, 18 declined consent and 14 were randomised to either SHC (n = 6) or ESDS (n = 8). The study profile is summarised in Figure 1. The most common reason for exclusion or non-recruitment was the inability to give informed consent. The full range of reasons for non-recruitment are shown in Table 2. Broadly these can be categorised into medical reasons (66%), social reasons (19%) and other reasons [‘missed’ or declined] (15%).

**Table 2:** Reasons for non-recruitment

***Reasons for non-recruitment***	**N**	**%**
Confusion (Abbreviated Mini-mental Test Score [AMTS] <7)	37	20
Require more complex multi-disciplinary team [MDT] input (physiotherapy, OT, social services)	35	19
Infective exacerbation of COPD [other services available]	20	11
Other co-morbidities requiring in patient stay	18	9.5
Clinical deterioration or mental health issues	17	9
Patient declined	13	7
Awaiting investigations to exclude pulmonary emboli	11	6
‘Missed’	10	5
Too well (suitable for discharge without support)	10	5
Carer/next of kin (NOK) declined	5	2.5
Too hypoxic	4	2
No respiratory infection	3	2
INR issues	3	2
Total	186	100

NB: multiple reasons may apply for the same patient.

### Patient consent

Of the remaining 32 eligible patients (after removal of n = 10 who were ‘missed at repeat review’), 14 patients consented to participation, 18 declined (see Figure 1). The demographics and clinical characteristics of those recruited and those who declined are shown in Table 3. Reasons given by patients for not wishing to consent included extra blood tests [n = 1], extra outpatient appointment [n = 1], ‘feel too unwell for home yet’ [n = 5] and other (‘not keen on research’, ‘steep stairs’, ‘daughter on holiday’) [n = 5].

**Table 3:** Demographics and characteristics of patients who declined or were recruited

	**Declined**		**Recruited**	
	**Patient (n = 13)**	**NOK (n = 5)**	**SHC (n = 6)**	**ESDS (n = 8)**
**Age (mean [range])**	66 [25 – 84]	79 [68 – 87]	70 [52 – 90]	61 [29 – 82]
**Gender (M:F)**	6 : 7	3 : 2	2 : 4	5 : 3
**Smoking status**	Not recorded		Ex – 3	Ex – 3
			Current – 2	Current – 2
			Never – 1	Never – 3
**Social history**	Live alone – 4	Live alone – 5	Live alone – 3	Live alone – 1
	With spouse – 6		With spouse – 2	With spouse – 5
	With family – 3			
			With family – 1	With family – 2
**CURB–65 (median [range])**	Not recorded		2 [1 – 3]	1 [0 – 2]
**Total hospital LOS (mean days [range])**			8.33 [1 – 31]	3.4 [1 – 7]
**New radiological consolidation**			Definite – 5	Definite – 4
			Possible – 0	Possible – 2
			None – 1	None – 2

Age, gender, smoking status and social history were recorded from screening data.

The mean age of recruited patients was 64.6 (29-90) yrs old; this was lower than in those whose NOK declined consent. Subjects were allocated a CURB-65 score whether or not consolidation was seen on their chest radiograph. New radiological consolidation was defined as definite, possible or none; this was decided by discussion between 2 respiratory clinicians. The median CURB-65 of all recruited patients was 1 (range 0-3), the majority of patients lived with spouse or family (72%) compared to all of those whose NOK declined who lived alone. The average time from admission to recruitment was 8 (1-9) days. Two recruited patients had positive microbiology – one *Haemophilus influenza* in sputum, the other *Streptococcus pneumoniae* in blood cultures.

### Safety and efficacy of intervention

Two subjects from SHC, and none from ESDS were readmitted (within 30 days) of discharge. There was 1 death in ESDS arm (known palliative lung cancer) and 1 death in SHC arm (aspiration pneumonia on readmission – possible underlying lung malignancy). The total LOS was 8.33 (1–31) days in SHC and 3.4 (1–7) days in the ESDS arm respectively. One subject from the SHC arm developed a presumed HAI. The maximum number of home visits needed was 4 (generally 1–3). The total length of stay in the ESDS was between 2–6 days. Subject and carer/NOK satisfaction (see table for example questions and scoring) was generally good.

Twelve subjects completed all SF-12 questionnaires (see Additional file 3) [day 0, 2, 7 and 28]. Overall mean increase of 0.4 points/subject was seen in SHC, and 1 point/subject in ESDS between day 0 and day 28. NB: using the SF-36 (a similar questionnaire with 36 questions) a 20-point change in the scale is believed to represent a clinically meaningful change; using SF-12 at least a 6-point change is deemed necessary for clinical significance).

With regards to symptom improvement, using CAP-SYM questionnaires,% recovery at day 28 (from baseline) could only be calculated in 3 SHC and 6 ESDS patients; with 88% and 90% recovery seen respectively at 28 days; therefore no difference between the 2 groups.

During the study, we collated a table of the common obstacles to recruitment (Table 4) that mainly refer to staff practice within the hospitals.

**Table 4:** Common obstacles to recruitment

Medical	• Pneumonia may be a vague diagnosis in hospital practice therefore large numbers of patients with respiratory infection need to be screened to find eligible patients
	• Lack of capacity to give consent
Staff	• Lack of physician ‘buy-in’ and resistance to change
Social	• Hospital stay may be seen as a respite opportunity for some carers
Patient belief	• Some patients believe that they must be 100% better before hospital discharge; some were suspicious of a new or research-based service.

## Discussion

We have shown that using defined criteria for recruitment and a defined interventional package, it is feasible for some patients with LRTI and pneumonia, who would otherwise have been treated in hospital, to be treated at home. Using our current model however large numbers of patients needed to be screened (n = 200) in order to recruit low numbers (n = 14). The ESDS package was successfully implemented in 7 patients with no adverse events. Randomisation was acceptable to patients and only deters those who *do not* wish to go home. The main obstacle to eligibility was lack of capacity to give informed consent. The number of eligible patients could be doubled if chronically confused or demented patients were included. Virtual visits (via telephone), rather than home visits may be adequate after the first 48 hrs after discharge.

The main strengths of this study are its novelty. We have been able to recognise common recruitment obstacles and find solutions to aid future project development. It has been noted previously in similar schemes that patient/carer refusal tends to reduce if a scheme becomes an adopted hospital service rather than a research project.

The weaknesses of this study are that it is a small feasibility study in a single city therefore no powered outcome data is available. The criterion of ‘requiring at least one more night of hospitalisation’ may be considered by some to be a weak criterion. The overall aim of the study is to reduce hospital bed days within a ‘real-life’ hospital setting in the UK. One more night of hospitalisation may be due to a variety of reasons and cannot simply be defined according to pre-defined signs or symptoms, as appropriate time for discharge for a patient with LRTI is physician-specific and no specific guidelines exist. We considered reasons that a patient would have ‘taken up’ a bed in hospital for at least one more night if ESDS were not available, these included: the need for further daily INR checks and low molecular weight heparin administration (with no facilities to have this performed immediately daily in the community), physician suggesting a further period of inpatient review for at least 24 hours after having changed from intravenous to oral antibiotics to ensure no pyrexia develops, no ability to get food supplies in at the patient’s home until the next day and insufficient ward staff to organise oxygen delivery and transport the same day, all leading to delayed discharge. All patients recruited received more intensive medical care than standard hospital care due to clinician sampling visits; this may affect the results of satisfaction questionnaires. Questionnaire data may be subject to recall bias. Also day 0 was defined as the day that the patient was deemed fit to be discharged home with support and not the first day of illness or day of admission; therefore this may not have captured the peak impact of the illness on their symptoms.

Our study, like previous studies of AA and ESDS for CAP and LRTI have shown recruitment may be difficult. In one study, 985 patients needed to be screened to find 214 eligible and 84 recruits, of which 53 had a diagnosis of CAP [23], in another 540 were screened to recruit 25 in each arm of study [24]. Low programme acceptance has been noted due to decline by physician (11%), patient (38%) or next of kin (36%) [25]. A study recruiting 55 patients with CAP in New Zealand in 2005 showed improved patient satisfaction by 40% (p < 0.001) and improved sleep but increased total days of care and no improvement in symptom score or function at 2 & 6 weeks [24]. Other studies have shown reduced bed days and hospitalisation (12% reduction) and overall cost reductions of $1489 and $(CAN)1016 [23,26].

We have previously shown in a retrospective study where two reviewers used pre-defined inclusion and exclusion criteria to assess eligibility to an ESDS, 48% of patients were deemed suitable for early supported discharge. The mean age of patients was 70 yrs old (range 18–96), 58% CURB-65 ≤ 2 and co-morbidities were common; COPD (30%) and dementia (15%). The total potential reduction in length of stay was calculated at 2.75 (range 1–7) days; amounting to a potential saving of 687,500 bed days annually in England [27].

The potential patient-related (reduced risk of HAI, care in own home, improved sleep, increased recovery rate, improved patient and carer satisfaction, reduced risk of delirium and later post-hospital discharge institutionalisation) and health-service benefits (reduced risk of HAI, improved self-management, reduced hospital LOS and therefore cost) are critical in assessing service impact. Strategies to increase the proportion of low-risk patients with CAP treated in the community have been developed and have been reported as safe, effective and acceptable to patients [28]. There is an urgent need for more evidence regarding ESDS to facilitate the discharge of patients with more complicated needs, due to the increasing bed pressures on acute hospital trusts. A significant number of patients who have complex social/mental health needs or co-morbidities will however still require inpatient care.

Future developments to our model may include accepting patients in whom clear decisions have been made that no escalation in care is appropriate if after 48 hrs no improvement is seen as terminal care may be more appropriately delivered at home [29]. Accepting patients on IV antibiotics and developing closer links with ‘early response teams’ in order to facilitate fast and effective discharge of more complex patients may be useful, as the numbers of hospital beds reduce in the UK [11].

We interpret our data to indicate that to improve recruitment future study directions should include: (1) *Hospital logistics* - working with hospital management to improve hospital systems to reduce time spent screening ineligible patients, increasing recruitment hours up to 12 hours-per-day, 7-days-per-week and improved ESDS ‘marketing’ (2) *Medical conditions* - the use of consultee declarations and retrospective consent allowing recruitment of suitable patients who lack capacity (3) *Staff* - improving physician education with regards to pneumonia and LRTI diagnosis and PE risk in order to reduce over-investigation/defensive practice, better study and clinical team integration (knowledge that the study team can reduce the team’s workload by facilitating discharge and conducting out-patient appointments) thereby decreasing physician refusal and earlier patient contact with the study team, enabling closer relationships to be formed thereby reducing the likelihood of ‘mixed messages’.

We estimate that by implementing the various methods described to overcome barriers to recruitment we could improve recruitment by 37.5%.

## Conclusion

In conclusion, an ESDS is difficult but not impossible to implement. Large numbers are needed to effectively assess safety and effectiveness. HAH care is a complex clinical model [30] that may work best as part of a portfolio of models (both AA and ESDS) promoting the for patients with respiratory infection [31]. HAH presents an opportunity to improve health policy, healthcare delivery and services; and to reduce admission rates and HAIs, all areas of major strategic importance internationally. We propose a large RCT with multiple relevant patient-related end-points is urgently needed [27].

## Abbreviations

ADL: Activities of daily living; AKD: Acute kidney disease; AMTS: Abbreviated mini-mental test score; CCF: Congestive cardiac failure; CKD: Chronic kidney disease; COPD: Chronic obstructive pulmonary disease; CRP: C-reactive protein; EWS: Early warning score; GCS: Glasgow coma scale; HR: Heart rate; IHD: Ischaemic heart disease; INR: International normalised ratio; MI: Myocardial infarction; NSTEMI: Non-ST elevation myocardial infarction; PU: Pass urine; RR: Respiratory rate; (S)BP: (Systolic) blood pressure; TnI: Troponin I; WCC: White cell count.

## Competing interests

AC - is funded by The Bill and Melinda Gates Foundation, and has no potential conflicting interests. She has received funds from Merck (MSD) and GSK for lecture fees and to attend conferences.

OE - no potential conflicting interests.

CH - no potential conflicting interests.

DG - NIHR Doctoral Research Fellowship, and has no potential conflicting interests.

SG - has no potential conflicting interests. He has received funds from Merck (MSD) and Novartis to attend conferences.

## Authors’ contributions

AC - made substantial contributions to the study conception and design, acquisition, analysis and interpretation of data, manuscript drafting and revision; and has given final approval of the version to be published. OE - made substantial contributions to data acquisition and manuscript revision; and has given final approval of the version to be published. CH - made substantial contributions to data collation and manuscript revision; and has given final approval of the version to be published. DW - made substantial contributions to the initial study concept, data acquisition and manuscript revision; and has given final approval of the version to be published. SG - made substantial contributions to the study conception and design, data interpretation, manuscript drafting and manuscript revision; and has given final approval of the version to be published. All authors read and approved the final manuscript.

## Pre-publication history

The pre-publication history for this paper can be accessed here:

http://www.biomedcentral.com/1471-2466/14/25/prepub

## Supplementary Material

Additional file 1 Satisfaction (Patient and Carer) Survey Questionnaire. Click here for file

Additional file 2 Emergency Patient Information Leaflet – lists red flag symptoms and contact numbers, leaflet given to all patients in the ESDS arm. Click here for file

Additional file 3 SF-12 (Functionality) Questionnaire. Click here for file

## References

[B1] Goss CH Rubenfeld GD Park DR Sherbin VL Goodman MS Root RK Cost and incidence of social comorbidities in low-risk patients with community-acquired pneumonia admitted to a public hospital Chest 2003 124 6 2148 2155 10.1378/chest.124.6.2148 14665494

[B2] Wennberg JE Freeman JL Culp WJ Are hospital services rationed in New Haven or over-utilised in Boston? Lancet 1987 1 8543 1185 1189 2883497 10.1016/s0140-6736(87)92152-0

[B3] Wennberg JE McPherson K Caper P Will payment based on diagnosis-related groups control hospital costs? N Engl J Med 1984 311 5 295 300 10.1056/NEJM198408023110505 6429534

[B4] Lim WS Woodhead M British thoracic society adult community acquired pneumonia audit 2009/10 Thorax 2011 66 6 548 549 10.1136/thoraxjnl-2011-200081 21502103

[B5] Arnold FW Ramirez JA McDonald LC Xia EL Hospitalization for community-acquired pneumonia: the pneumonia severity index vs clinical judgment Chest 2003 124 1 121 124 10.1378/chest.124.1.121 12853513

[B6] Masotti L Ceccarelli E Cappelli R Barabesi L Guerrini M Forconi S Length of hospitalization in elderly patients with community-acquired pneumonia Aging (Milano) 2000 12 1 35 41 10746430 10.1007/BF03339826

[B7] Choudhury G Chalmers JD Mandal P Akram AR Murray MP Short P Singanayagam A Hill AT Physician judgement is a crucial adjunct to pneumonia severity scores in low-risk patients Eur Respir J 2011 38 3 643 648 10.1183/09031936.00172910 21406507

[B8] Welte T Torres A Nathwani D Clinical and economic burden of community-acquired pneumonia among adults in Europe Thorax 2012 67 1 71 79 doi: 10.1136/thx.2009.129502. Epub 2010 Aug 20 10.1136/thx.2009.129502 20729232

[B9] Raut M Schein J Mody S Grant R Benson C Olson W Estimating the economic impact of a half-day reduction in length of hospital stay among patients with community-acquired pneumonia in the US Curr Med Res Opin 2009 25 9 2151 2157 10.1185/03007990903102743 19601711

[B10] Torres OH Munoz J Ruiz D Ris J Gich I Coma E Gurgui M Vazquez G Outcome predictors of pneumonia in elderly patients: importance of functional assessment J Am Geriatr Soc 2004 52 10 1603 1609 10.1111/j.1532-5415.2004.52492.x 15450034

[B11] Shepperd S Iliffe S Hospital at home versus in-patient hospital care Cochrane Database Syst Rev 2005 3 CD000356 10.1002/14651858.CD000356.pub2 16034853

[B12] Goldberg AI Faure EA Home care for life-supported persons in England. The responaut program Chest 1984 86 6 910 914 10.1378/chest.86.6.910 6437752

[B13] Davies L Wilkinson M Bonner S Calverley PM Angus RM “Hospital at home” versus hospital care in patients with exacerbations of chronic obstructive pulmonary disease: prospective randomised controlled trial BMJ 2000 321 7271 1265 1268 10.1136/bmj.321.7271.1265 11082090 PMC27532

[B14] Davison AG Monaghan M Brown D Eraut CD O'Brien A Paul K Townsend J Elston C Ward L Steeples S Cubitt L Hospital at home for chronic obstructive pulmonary disease: an integrated hospital and community based generic intermediate care service for prevention and early discharge Chron Respir Dis 2006 3 4 181 185 10.1177/1479972306070074 17190120

[B15] Ojoo JC Moon T McGlone S Martin K Gardiner ED Greenstone MA Morice AH Patients’ and carers’ preferences in two models of care for acute exacerbations of COPD: results of a randomised controlled trial Thorax 2002 57 2 167 169 10.1136/thorax.57.2.167 11828049 PMC1746235

[B16] Aimonino Ricauda N Tibaldi V Leff B Scarafiotti C Marinello R Zanocchi M Molaschi M Substitutive “hospital at home” versus inpatient care for elderly patients with exacerbations of chronic obstructive pulmonary disease: a prospective randomized, controlled trial J Am Geriatr Soc 2008 56 3 493 500 10.1111/j.1532-5415.2007.01562.x 18179503

[B17] Cotton MM Bucknall CE Dagg KD Johnson MK MacGregor G Stewart C Stevenson RD Early discharge for patients with exacerbations of chronic obstructive pulmonary disease: a randomized controlled trial Thorax 2000 55 11 902 906 10.1136/thorax.55.11.902 11050257 PMC1745631

[B18] Wilson A Parker H Wynn A Jagger C Spiers N Jones J Parker G Randomised controlled trial of effectiveness of Leicester hospital at home scheme compared with hospital care BMJ 1999 319 7224 1542 1546 10.1136/bmj.319.7224.1542 10591717 PMC28299

[B19] Nicholson C Bowler S Jackson C Schollay D Tweeddale M O’Rourke P Cost comparison of hospital- and home-based treatment models for acute chronic obstructive pulmonary disease Aust Health Rev 2001 24 4 181 187 10.1071/AH010181 11842709

[B20] Ewig S Welte T Chastre J Torres A Rethinking the concepts of community-acquired and health-care-associated pneumonia Lancet Infect Dis 2010 10 4 279 287 10.1016/S1473-3099(10)70032-3 20334851

[B21] Jenkinson C Layte R Jenkinson D Lawrence K Petersen S Paice C Stradling J A shorter form health survey: can the SF-12 replicate results from the SF-36 in longitudinal studies? J Public Health Med 1997 19 2 179 186 10.1093/oxfordjournals.pubmed.a024606 9243433

[B22] Lamping DL Schroter S Marquis P Marrel A Duprat-Lomon I Sagnier PP The community-acquired pneumonia symptom questionnaire: a new, patient-based outcome measure to evaluate symptoms in patients with community-acquired pneumonia Chest 2002 122 3 920 929 10.1378/chest.122.3.920 12226033

[B23] Frick KD Burton LC Clark R Mader SI Naughton WB Burl JB Greenough WB Steinwachs DM Leff B Substitutive hospital at home for older persons: effects on costs Am J Manag Care 2009 15 1 49 56 19146364

[B24] Richards DA Toop LJ Epton MJ McGeoch GR Town GI Wynn-Thomas SM Dawson RD Hlavac MC Werno AM Abernethy PD Home management of mild to moderately severe community-acquired pneumonia: a randomised controlled trial Med J Aust 2005 183 5 235 238 16138795 10.5694/j.1326-5377.2005.tb07026.x

[B25] Santos-Eggimann B Chavaz N Larequi T Lamy O Yersin B Heart failure and community-acquired pneumonia: cases for home hospital? Int J Qual Health Care 2001 13 4 301 307 10.1093/intqhc/13.4.301 11560349

[B26] Loeb M Carusone SC Goeree R Walter SD Brazil K Krueger P Simor A Moss L Marrie T Effect of a clinical pathway to reduce hospitalizations in nursing home residents with pneumonia: a randomized controlled trial JAMA 2006 295 21 2503 2510 10.1001/jama.295.21.2503 16757722

[B27] Collins AM Wilks S Wootton D Gordon SB Supported home-care schemes: the key to increasing outpatient care? Eur Respir J 2012 39 2 508 10.1183/09031936.00165911 22298620

[B28] Chalmers JD Akram AR Hill AT Increasing outpatient treatment of mild community-acquired pneumonia: systematic review and meta-analysis Eur Respir J 2011 37 858 864 10.1183/09031936.00065610 20729221

[B29] Shepperd S Wee B Straus SE Hospital at home: home-based end of life care Cochrane Database Syst Rev 2011 7 CD009231 10.1002/14651858.CD009231 PMC4033790 21735440

[B30] Campbell M Fitzpatrick R Haines A Kinmonth AL Sandercock P Spiegelhalter D Tyrer P Framework for design and evaluation of complex interventions to improve health BMJ 2000 321 7262 694 696 10.1136/bmj.321.7262.694 10987780 PMC1118564

[B31] Leff B Defining and disseminating the hospital-at-home model CMAJ 2009 180 2 156 157 10.1503/cmaj.081891 19153385 PMC2621275

