# Supplementary material for: Feasibility study for early supported discharge in adults with respiratory infection in the UK
Source: BMC Pulm Med. 2014 Feb 26;14:25. doi: 10.1186/1471-2466-14-25 (PMC3943804; doi:10.1186/1471-2466-14-25)
Supplement: Additional file 1 — Satisfaction (Patient and Carer) Survey Questionnaire. [file 1471-2466-14-25-S1.docx]

***Additional file 1***: **Satisfaction (Patient and Carer) Survey Questionnaire.**

| Q1 | I have been treated with kindness and respect by staff | Strongly agree (SA) | Agree (A) | Disagree (D) | Strongly disagree (SD) |
| --- | --- | --- | --- | --- | --- |
| Q2 | The staff attended well to my personal needs | Strongly agree (SA) | Agree (A) | Disagree (D) | Strongly disagree (SD) |
| Q3 | I was able to talk to the staff about any problems that I might have had | Strongly agree (SA) | Agree (A) | Disagree (D) | Strongly disagree (SD) |
| Q4 | I received all the information I wanted about the cause and nature of my illness | Strongly agree (SA) | Agree (A) | Disagree (D) | Strongly disagree (SD) |
| Q5 | The doctors and nurses have done everything they can to make me well again | Strongly agree (SA) | Agree (A) | Disagree (D) | Strongly disagree (SD) |
| Q6 | I am happy with the amount of recovery I have made | Strongly agree (SA) | Agree (A) | Disagree (D) | Strongly disagree (SD) |
