# Supplementary material for: Feasibility study for early supported discharge in adults with respiratory infection in the UK
Source: BMC Pulm Med. 2014 Feb 26;14:25. doi: 10.1186/1471-2466-14-25 (PMC3943804; doi:10.1186/1471-2466-14-25)
Supplement: Additional file 2 — Emergency Patient Information Leaflet – lists red flag symptoms and contact numbers, leaflet given to all patients in the ESDS arm. [file 1471-2466-14-25-S2.docx]

***Additional file 2***: **Emergency Patient Information Leaflet** – lists red flag symptoms and contact numbers, leaflet given to all patients in the ESDS arm

the ESDS arm.

**HOME FIRST**

Do you have any of the following?

List of symptoms to prompt contact

- **Fever (>38 ^˚^C)**
- **Increasing drowsiness**
- **Worsening cough or sputum**
- **Coughing up blood**
- **Increasingly unwell**
- **Feeling faint**
- **Vomiting – unable to keep antibiotics down**

[HOME Followed-up by Infection Respiratory Support Team]


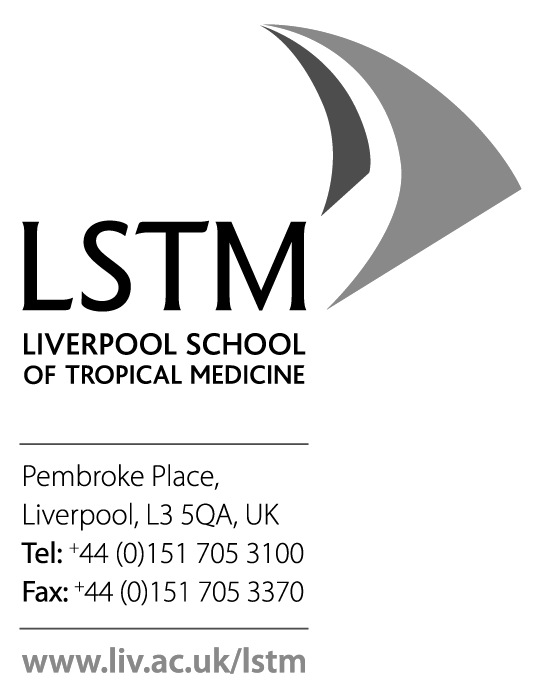


Patient Information Sheet

**EMERGENCY CONTACT**

**YES**

**Direct to HOME FIRST research team weekdays**

**xxxxxxxxxxxx**

**Call HOME FIRST**

**Direct to HOME FIRST research team weekdays xxxxx**

**Outside office hours (24hr emergency contact) xxxxx and ask for the on-call HOME FIRST Doctor**

**OR**

**Attend AED/call 999**

**At all other times phone**

**xxxxxx**

**Hospital Switchboard**

**Ask for the on-call HOME FIRST Doctor**

What should I do?

You should also contact the HOME FIRST research team immediately.

**Outside office hours** - ask the Hospital switchboard for the **on-call HOME FIRST Doctor**. They will be available by telephone 24-hours-a-day for advice.

## What if I feel very unwell?

In the event you feel very unwell we advise you to immediately phone the emergency research team. Or alternatively in the event of an emergency attend the Emergency Department, NHS Walk-in centre or call 999.

## What do I tell the doctor?

If, for any reason you have to attend your doctor or the hospital you need to inform them that:

You have .......................................................................... and are being cared for at home by HOME FIRST – a supported early discharge team for patients with respiratory infection as part of a research study.

# Things you should know........

Discharge home

You will be transferred home using a hospital taxi and accompanied by the study nurse. All your discharge medications will be dispensed by pharmacy prior to discharge. Meals-on-wheels will be arranged as needed. You may have been provided with a lifeline (pendant alarm) +/- home portable observations machine.

Follow-up

You must complete your antibiotic course as directed by the HOME FIRST staff. Your patient visit diary will list all the times & dates when the HOME FIRST team will visit your home. Additional dates may be arranged as needed, depending on how quickly you are recovering. You will also have clinic appointments at 1 and 6 months after discharge from hospital.

What should I look out for?

If you feel generally unwell or have any of the following:

- **Fever (>38 ^˚^C)**
- **Increasing drowsiness**
- **Worsening cough or sputum**
- **Coughing up blood**
- **Increasingly unwell**
- **Feeling faint**
- **Vomiting – unable to keep antibiotics down**
