# Supplementary material for: Feasibility study for early supported discharge in adults with respiratory infection in the UK
Source: BMC Pulm Med. 2014 Feb 26;14:25. doi: 10.1186/1471-2466-14-25 (PMC3943804; doi:10.1186/1471-2466-14-25)
Supplement: Additional file 3 — SF-12 (Functionality) Questionnaire. [file 1471-2466-14-25-S3.docx]

***Additional file 3*: SF-12 (Functionality) Questionnaire.**

| Q1 | In general would you say your health is? | 1 Excellent | 2 Very good | 3 Good | 4 Fair | 5 Poor | 6 Never |
| --- | --- | --- | --- | --- | --- | --- | --- |
| Q2 | Does your health now limit you in moderate activities i.e. moving a table, pushing a vacuum, bowling or playing golf | 1 A lot | 2 A little | 3 Not limited |  |  |  |
| Q3 | Limited climbing several flights of stairs | 1 A lot | 2 A little | 3 Not limited |  |  |  |
| Q4 | During the last 4 weeks have you had any problems with your work or regular activities as a result of your physical health and accomplished less than you would like to? | 1 YES | 2 NO |  |  |  |  |
| Q5 | During the last 4 weeks have you had any problems with your work or regular activities as a result of you physical health and been limited in the kind of work or other activities? | 1 YES | 2 NO |  |  |  |  |
| Q6 | During the past 4 weeks were you limited in the kind of work you do or other regular activities as a result of any emotional problems i.e. depressed / anxious and accomplished less? | 1 YES | 2 NO |  |  |  |  |
| Q7 | During the past 4 weeks were you limited in the kind of work you do or other regular activities as a result of emotional problems such as being depressed or anxious and didn’t do work or other activities as carefully as usual | 1 YES | 2 NO |  |  |  |  |
| Q8 | During the past four weeks how much did pain interfere with your normal work (including both work the home and housework) inside and outside | 1 Not at all | 2 A little bit | 3 Moderately | 4 Quite a bit | 5 Extremely |  |
| Q9 | How much of the time in the past four weeks have you felt calm and peaceful | 1 All of the time | 2 Most of the time | 3 A good bit of the time | 4 Some of the time | 5 A little of the time | 6 None of the time |
| Q10 | How much of the time during the past four weeks did you have a lot of energy | 1 All of the time | 2 Most of the time | 3 A good bit of the time | 4 Some of the time | 5 A little of the time | 6 None of the time |
| Q11 | How much of the time during the past four weeks have you felt downhearted and blue | 1 All of the time | 2 Most of the time | 3 A good bit of the time | 4 Some of the time | 5 A little of the time | 6 None of the time |
| Q12 | During the past four weeks, how much of the time has your physical health or emotional problems interfered with social activities (like visiting with friends, relatives etc.) | 1 All of the time | 2 Most of the time | 3 A good bit of the time | 4 Some of the time | 5 A little of the time | 6 None of the time |
